# Older adults’ experiences of taking up a new community-based leisure activity to promote brain health: A focus group study

**DOI:** 10.1371/journal.pone.0290623

**Published:** 2023-09-11

**Authors:** Malwina A. Niechcial, Calum Marr, Lauren M. Potter, Adele Dickson, Alan J. Gow

**Affiliations:** Department of Psychology and Centre for Applied Behavioural Sciences, School of Social Sciences, Heriot-Watt University, Edinburgh, United Kingdom; School of Health Binawan: Universitas Binawan, INDONESIA

## Abstract

**Introduction:**

An active and engaged lifestyle is supported as being beneficial for brain health. Activities comprising physical, mental and social demands, or combinations of those, are of particular interest, and have been the focus of specific interventions. Exploring how older people engage with such community-based activities, including facilitators and barriers to participation, may help improve the success of future translational activities. The purpose of this study was therefore to identify factors that enabled or hindered activity engagement by conducting focus groups with people who had been supported to take up a new activity as part of an intervention study.

**Materials and methods:**

Twenty-seven older adults aged 65–86 (56% female) who had completed an activity-based intervention study participated in three focus groups. Discussions explored their experiences of taking up a new activity, including facilitators and barriers to their engagement, and their perceptions of any benefits.

**Results:**

Thematic analysis grouped participants’ responses into five themes: positive aspects and facilitators of engagement in a new activity; challenges and barriers to engagement; ageing being a facilitator and a barrier to engagement; differential effects of activities on participants’ health and wellbeing; and general project feedback (including opinions on study design).

**Discussion and conclusions:**

Participants’ experiences and expectations included positive (e.g., enjoyment, socialisation) and negative factors (e.g., lack of confidence, other commitments, class costs and poor structure), consistent with previous research on social participation and engaging with new learning opportunities. Future studies should also consider those who do not readily participate in leisure activities to address earlier barriers. It is important that older adults have access to potentially beneficial activities and local authorities should prioritise increasing their provision.

## Introduction

Changes in our cognitive abilities as we age are influenced by our lifestyles. An active and engaged lifestyle is supported as being beneficial for brain health [1–4]. Engaging in activities in mid- or later life may be associated with lower risk of dementia [5,6]. Having a more open disposition may be related to increased engagement in mentally challenging activities and better cognition [7].

Real-world activities varying in their levels and combinations of physical, mental and social demands, are gaining increasing support as potential multimodal cognitive interventions. For example, Park and colleagues [3] studied the effects of engagement in *novel* activities (where participants reported no or limited prior exposure) on the cognitive abilities of older adults. Participants engaging in productive activities (requiring new learning) for 12 weeks showed improvements in episodic memory, with limited cognitive benefits observed in the receptive condition (not requiring new learning). In another study, older tablet computer novices took part in 10 weeks of learning to use the new technology [8]. The training group experienced a significant increase in processing speed versus no contact controls [8]. In post-intervention focus groups, conducted before the quantitative analyses had been completed, participants specifically mentioned feeling quicker and thinking faster as a result of their tablet training [9].

Activity participation may produce benefits that reach beyond cognitive improvements. Namely, they can promote aspects of general health and wellbeing [10], such as enhanced quality of life, socialisation and functional health [11]. It is possible that those who are lonely and do not generally take part in such activities may experience greater benefits than those reported in generally active samples [12].

That leisure activities may support older adults’ thinking skills and aspects of wellbeing is valuable. However, many older adults do not readily participate in learning programmes, including those designed with older participants in mind, such as the University of the Third Age (U3A). For example, while many Iranian older adults participate in religious activities (62.4% said they often or always went to religious places), 86.5% never or seldom participate in group or individual exercise, with 99.3% never/seldom participating in educational classes [13]. Similarly, 13% of respondents aged 57–76 in an English survey were taking part in educational classes and 25% in some form of exercise [11]. In another study, only two percent of retired participants (aged 65 and over) took part in formal learning (leading to a qualification), with another two percent engaging in informal learning (not leading to a qualification; [10]). These figures show low levels of engagement in different types of activities among older adults of different backgrounds. It is, therefore, important to consider what may boost or hinder participation in those activities.

Socioemotional Selectivity Theory (SST; [14]) argues that older adults seek activities that bring joy and meaning as they may perceive time to be limited. However, this is unlikely to include new learning as exploration and gaining knowledge may not be as important as emotional satisfaction in later life [15]. Low engagement in diverse learning programmes may also be influenced by more specific factors. These can include factors that may be considered internal (lack of knowledge and confidence, feelings of inadequacy, negative comparisons with younger people, embarrassment, previous negative school experiences) or external (lack of time due to other commitments, high costs, inadequate or difficult to obtain information, limited local provision and transport) [16–19]. Social factors, such as not having family and friends (or others in the class) to encourage and support learning may also inhibit participation [20]. People may also be prone to holding negative ageing stereotypes [21,22], which may have tangible negative effects on older adults’ health and wellbeing [23]. Those stereotypes may also prevent older adults from engagement in new things, such as technology use [24]. To encourage participation in beneficial activities, it is important to more fully understand why older adults may not engage with leisure activities and learning opportunities, especially because the factors may be varied [17] and may depend on culture, location, personal circumstances and more practical things, like provision and transport.

The Intervention Factory study, the setting for the current focus groups, was specifically designed to examine how engagement in community-based activities might benefit the health and wellbeing of healthy older adults aged 65 and over. Community-based activities were chosen to break down the inherent translational barrier of interventions designed specifically for research studies (such interventions are “artificial” and not reflective of the real world). Following their participation, participants were invited to focus groups to evaluate the study. In addition to providing insights on the suitability and acceptability of the study design, the focus group methodology was chosen to generate a deeper understanding of individual beliefs and attitudes [25], in this case regarding factors affecting taking up a new activity in later life.

The current study examined the experiences of healthy older adults taking up a new activity as part of the Intervention Factory study to address the following questions:

- How do older adults view their participation in the Intervention Factory study and the new activities taken up as part of the study?
- To what extent do they think those new activities improved their cognitive abilities and other aspects of their general health and wellbeing?
- What do they perceive to be factors affecting participation in a new activity more broadly?

## Materials and methods

### Participants

The Intervention Factory study examined how engagement in novel, community-based activities might benefit the health and wellbeing of older adults aged 65 and over. We selected activities based on mental (e.g., computer classes), physical (e.g., exercise classes, Zumba, line dancing) and social engagement (e.g., social groups, bingo), and combinations of these (e.g., creative activities, such as pottery, woodwork, stained glass, printmaking, and language classes). The fuller details of the Intervention Factory study including the protocol and the quantitative data are beyond the scope of this paper and will be published separately.

Briefly, three hundred and thirty-six generally healthy community-dwelling older adults aged 65 and over participated. Participants were excluded if they had a diagnosis of neurodegenerative conditions, stroke, brain tumour, head injury, significant manual impairment. Participants were also ineligible if they had prior experience in all of the activities offered as interventions, and if they lived outside of the study recruitment area and were unable to travel to the area regularly (for fuller sample details including recruitment strategies see [26]. Participants completed baseline testing, were allocated to a new activity for 10–12 weeks, then returned for follow-up testing upon completion of their activity. Of the 302 who completed follow-up testing, 92 took up a second activity, with 80 returning for their second follow-up testing. Twenty-seven participants (who had completed one or two activities during the intervention study) took part in focus groups. Participants shared their experiences of the activities they completed as part of the Intervention Factory study, including facilitators and barriers of taking part in those new activities (Table 1). Participants were asked to describe their gender and ethnicity in their own words during baseline testing. This information was only used to describe the sample.

**Table 1:** Sample characteristics.

Variable	N (%)	Mean	SD	Min	Max
Gender Female Male	15 (44) 12 (56)				
Age		71.8	5.4	65	86
Ethnicity White English/Welsh/Scottish/Northern Irish/British European	5 (19) 19 (70) 3 (11)				

### Design and procedure

Focus group size was based on previous literature citing the number of participants between six and 10 as ideal [27,28]. Nine participants were included in each of three groups to cover the variety of activities people took up as part of the Intervention Factory study. Recruitment to the focus groups stopped once data saturation has been reached.

We conducted focus groups in a meeting room at Heriot-Watt University, Edinburgh in November 2019, with participants and moderator (lead author, M.A.N.) seated around a table. Questions were adapted from a previous study [9]. For the current study, questions focused on participants’ experiences of taking up a new activity under the auspices of the Intervention Factory study. That also included reflecting on the facilitators and barriers of taking up new activities more broadly. Each group discussion lasted approximately 1.5hrs. Discussions were audio and video recorded with participants’ consent, and subsequently transcribed verbatim.

Firstly, the moderator reminded the participants about the aims of the focus groups. The focus groups explored participants’ opinions of their activities, whether they thought the activity improved their thinking skills and general health and wellbeing, the perceived factors affecting taking up a new activity more generally, and any other study feedback. The complete list of questions in the semi-structured agenda are in S1 Appendix (Supplementary material). To ensure all participants had a chance to contribute to the discussion, the moderator would look around the room to give gentle encouragement to less responsive participants to speak by making eye contact while asking a question or checking whether anyone else had something to say. The moderator did not directly address specific participants to avoid unnecessary pressure to respond.

This study was approved by the Heriot-Watt University School of Social Sciences Ethics Committee (Ref: 2017–453). All participants gave written informed consent prior to taking part in accordance with the Declaration of Helsinki.

### Data analysis

The focus groups were transcribed verbatim and subjected to thematic analysis as described by Elo and Kyngäs [29]. Analysis was inductive in nature, where categories were derived from the data being studied.

We conducted the thematic analysis using NVivo 12. The lead author read the transcripts in detail, and then coded and categorised data from the first focus group. To counteract the risk of bias, the second author (C.M.) independently read the same transcript and coded and categorised the data. The resulting codes were then compared for agreement. Where the codes differed (wording used to describe a code or diverging concepts), the authors discussed the issue, explaining the reasons for their codes until a consensus was reached. Following the discussion, the coding template was updated, and the lead author used it to code the rest of the transcripts. The data were continuously reviewed to refine the codes when new information required additional or more sophisticated codes. The lead author selected an illustrative quote for each of the codes and read, and re-read the list of codes multiple times, condensing the list where two or more codes covered similar concepts and would have benefitted from being merged. Once the lead author was satisfied with the number and content of codes, she then grouped similar concepts together, resulting in sub-themes. Grouping the sub-themes continued until she was satisfied with the emergent themes. The lead author then re-evaluated the content of each theme until she was satisfied that the themes were an accurate reflection of what was discussed in the focus groups.

## Results

Participants described positive and negative aspects of experiences of the study and taking up a new activity more broadly, and identified specific effects the activities may have had on their wellbeing. The participants also discussed ageing as a facilitator and a barrier to participation in leisure activities, and gave their views on study design. Thematic analysis of the focus group transcripts revealed five distinct themes relating to participants’ experiences of taking up a new activity (Table 2). The five themes will be described further below, labelled: *Positive aspects and facilitators of engagement in a new activity; Challenges and barriers to engagement in a new activity; Ageing as a facilitator and a barrier to engagement in a new activity; Differential effects of activities;* and *General project feedback and considerations*.

**Table 2:** Themes and sub-themes in participants’ experiences of, and opinions about, taking up a new activity.

Theme	Sub-theme	Quotes
Positive aspects and facilitators of engagement in a new activity	a) Positive aspects b) Socialisation c) Attendance boosters/Facilitators of engagement d) Motivation to continue/Facilitators of further engagement	*I’ve never done anything like that in my life and I enjoyed every minute of it*. (P1G3) *So*, *I think it was the social side for me*, *I mean*, *I did like doing woodwork as well but I looked forward to it from the*, *sort of*, *social side of being with*, *sort of*, *different people*. (P8G1) *I looked forward to mine and I’m a bit like you (looking at P1G2)*, *if I commit to something and say “I’ll do it” then I will*, *I’ll do it over the number of weeks I said I would*. (P2G2) *Honestly*, *it’s given me an interest in things that I never*, *ever had before*, *so that’s a*, *sort of*, *side effect that I’m really enjoying*. *And I really feel I will go back and take that up*. (P1G3)
Challenges and barriers to engagement in a new activity	a) Challenges of activities b) Factors hindering attendance/Barriers to engagement c) Drawbacks for continuing/Barriers to further engagement d) Barriers to taking up a new activity	*So*, *I decided after the first couple of weeks I was going to lower the bar because I just found it*, *I couldn’t get anything finished because there was*, *it was too difficult*, *I suppose*, *too new*. (P2G3) *I did Pottery as my second activity*, *which I would never in a million years have chosen and it was only because I was safe there that I went and everybody in the class was from the project*. (P2G2) *But as to continuing it*, *I mean* … *Unless you were taking it seriously*, *you’d have to invest in a lot of expensive glass and equipment*. *And so*, *it’s not something to take up at home*, *you definitely need to go to a class*, *you know*. (P3G1) *I think*, *I mean*, *I’ve always been awful at foreign languages and awful at dancing*, *you know*, *so it’s challenging yourself just to start and be embarrassed […] and all that*, *I think it’s the biggest challenge for me*. (P1G2)
Ageing as a facilitator and a barrier to engagement in a new activity	a) Benefits of ageing b) Challenges of ageing	*It’s quite nice as well when you get to the end and it’s cheaper because you’re older (laughter)*. *I quite like that*! (P8G1) *The challenge I faced was trying to remember all that had been said to me*, *even by the time I got home quickly just trying to scribble it down and make sense of it all*. (P1G1)
Differential effects of activities	a) Benefits of activity b) No change in abilities	*And I honestly felt*, *round about December time*, *I honestly felt that*, *it might sound silly*, *but I felt that my brain was working better*, *I really do*, *you know*, *felt*, *sort of*, *a wee bit sharper*, *you know*, *so*. (P3G3) *I think maybe because*, *it’s only like two hours a week for six weeks*, *and it’s such a small percentage of my time*, *I can’t really see that that’s had this big impact…* (P7G2)
General project feedback and considerations	a) Opinions on activities and tutors b) Challenges of adult education classes c) Benefits of adult education classes d) Project feedback	*I found it surprisingly challenging*, *you know*. *I’m quite*, *you know*, *I knit and I sew and I do bits and pieces but I’ve never done anything like that before and I found it a whole different language*. (P2G3) *I spent an awful lot of time like that but Council (local authority) classes are like that*. *They don’t chuck out the ones that are over-experienced or the class won’t have enough numbers to exist*, *so it made it harder*. (P5G1) *It was easy to get to*. *I didn’t know there was a Tollcross Community Centre*, *so I’ve discovered a new location that I could easily get to*. (P8G2) *Yeah*, *so again*, *I suppose I’d often thought it’d be quite nice to learn another language but never quite had the oomph and the courage […] to do it*, *whereas this sort of focused my mind*, *thinking “actually it’s not impossible to go on and do it*.*”* (P2G1)

### Positive aspects and facilitators of engagement in a new activity

The first theme consisted of positive aspects of taking up a new activity, specifically in relation to participants’ experiences with this study but also more broadly.

#### Positive aspects

Most participants mentioned their enjoyment, even if sometimes they did not think they would like the activity they were allocated to or found the activity challenging. Participants who took part in creative activities also mentioned feeling accomplished and proud of their creations, providing a confidence boost.

*I finished my piece and it’s about this size (demonstrating)*, *it’s hanging up at the window for the world to see (laughter)*. *I feel like going out and stopping people (laughter)*: *“Do you see that thing in the window*?*”* (P7G3)

#### Socialisation

Another aspect of their activities that most participants enjoyed and sought out was socialisation with others, whether their own age or younger. Many participants felt rejuvenated by contact with younger people and felt it was a welcome change from only seeing people their own age, which they suggested could become monotonous.

*It was nice being with people*, *I think I was about the only golden oldie*, *I think*, *in the class*, *actually*. *Yeah*, *but a lot of people were young and students*. *It was nice mixing with people not of my age*, *sorry (laughter)*. *Yeah*, *just for once*, *it was nice*. (P6G1)

#### Attendance boosters/facilitators of engagement

Most participants reported positive factors encouraging them to keep attending their activity. For example, participants were motivated to attend because of their participation in the study, simply enjoying the activity or being “desperate to see their finished article”. There was also an element of fear of missing out spurring participants on, both in the sense of dreading having to catch up on what was missed but also in terms of looking forward to the next stages of learning.

*I fell out in the garden and managed to do my rotator cuff in my left arm*, *so I couldn’t move it at all*. *And I went back the next week*, *even though it wasn’t healed*, *and I sat there (demonstrating working with one hand) trying to do things because I did want to go back*, *I didn’t want to miss out on it*. (P9G3)

#### Motivation to continue/facilitators of further engagement

About half the participants felt the activity gave them the motivation to continue pursuing their studies. Some participants also found that their new activity surprised them, having never previously considered taking part in it, sometimes even resulting in them finding a new hobby.

*It’s a very popular course in fact*. *I did enjoy it that much that I’m still on it because I just carry on […] so this course has benefitted us in home improvement*, *and I’ve made the garden gate for myself and the window frames for the garage*. (P7G1)

### Challenges and barriers to engagement in a new activity

Although participants identified many positive aspects of their activity, they also spoke about specific challenges and barriers to participation more broadly.

#### Challenges of activities

Most participants identified challenges specific to their activities. Those taking part in language classes frequently raised the difficulties of language learning generally, but also specifically in older age. They voiced their frustration at the time and effort spent on memorising new vocabulary, which was very challenging. Those attending creative activities were often worried about the dangers of using specialist equipment, such as glass cutters and wood cutting machines. However, the initial fear and anxiety diminished as the participants got more practice with those “scary” techniques and pieces of equipment and were eventually replaced by feelings of pride and accomplishment.

*[…] and then suddenly it was “oh we’re gonna use this machine” and you know*, *sort of*, *you know*, *you got this big thick piece of wood and the thing comes out and makes a big hole and I think “my hand’s there*, *it’s gonna go straight through”*, *you know*. *[…] And I was*, *I felt a little bit proud of myself when I’d done things on the machines because I knew I was so frightened of the machines*. (P8G1)

#### Factors hindering attendance/barriers to engagement

Although participants generally did not report problems with attending their activities, a small number found it challenging because they felt they were not learning and wasting their time. Others pointed out that missing one class could be an excuse not to go back. One participant also said they only went along because their class was full of other study participants, and they “felt safe” there. Otherwise, a class full of strangers with no apparent immediate connection would have been a barrier to participation.

#### Drawbacks for continuing/barriers to further engagement

Around a half of participants also discussed specific drawbacks for continuing with their allocated activity. About a third of those who took up creative activities mentioned the expensive materials and tools they would have to invest in if they wanted to continue (all class costs and any required materials had been paid by the study), presenting a financial burden, especially for those whose pension would not allow for additional expenses. Others felt it was not something they could easily take up at home and would need to attend an organised group to take part. Some participants also mentioned their struggles with physical challenges of their allocated activity preventing them from further engagement.

#### Barriers to taking up a new activity

Most of the participants highlighted broader considerations when taking up something new, such as time constraints (especially for those still working or providing childcare) and the fact that it may be hard to fit things in because “everything is out there and it’s all wonderful”. Most participants also spoke about the challenges of going outside of one’s comfort zone and putting oneself in a vulnerable position, requiring effort and resilience.

*Yeah and in my family we call it “the first day of school syndrome” and it’s sort of*, *like*, *there’s no way to make the first day the second day*, *it’s got to start somewhere*, *it’s the fact that you feel nervous and that you don’t know what to expect and that you’re not sure how well you’re do*, *you’re gonna do*, *is*, *comes with trying something and showing up*. (P8G2)

About a quarter of participants said their experiences at school held them back from trying something new. Some participants mentioned they were keen to try those activities they had negative experiences with in the past to find out for themselves almost as an act of defiance, while others were worried by the thought of looming failure.

*Am I the only one that wouldn’t do something that might make you feel bad*? *Now*, *what does that say about me*? *But*, *you know*, *I had such bad experiences at school with languages*, *so I wouldn’t even*, *I’m not strong enough to even challenge me again*. (P4G3)

### Ageing as a facilitator and a barrier to engagement in a new activity

The findings presented thus far describe a range of positive and challenging aspects, including facilitators and barriers to engagement, varying between participants and the activities they undertook. Ageing was, however, suggested as either a facilitator or a barrier, highlighted by about three-quarters of our participants.

#### Benefits of ageing

Some participants appreciated the opportunities afforded to them by retirement, including the time they had to do “lots of things” because they were “free birds” now and they could do what they liked. This can be very important at an older age when people face less pressures from work and family, which may have taken priority before. Retirement is then seen as a time to take back some degree of independence and do things that are important for them and make them happy.

*I spent all my life working*, *so when I retired*, *which was not very long ago*, *I’ve opened up a whole new world in front of myself*. *I’m doing all the kinds of things which I wanted to do*. *[…] Because I have two priorities in my life*, *one is to keep myself physically active and keep myself mentally active*. *All the rest follows on from that*. (P6G1)

Three participants highlighted old age concessions, making participation in community-run classes a little cheaper, or free bus passes to help them get around as benefits of being older. The convenience of frequent bus services and free travel was something they appreciated, making them feel independent and empowered to live the life they want to, able to get anywhere they like without worrying about the cost.

Although some participants mentioned being self-conscious about their older bodies because they didn’t look like they used to or being anxious about “making a fool out of themselves”, especially compared to younger people, one participant made a comment that felt very empowering. Instead of negatively comparing herself to young adults, she said:

*Well*, *my attitude is different*, *actually*, *because I think when I was younger*, *I was trying (not) to make a mistake and maybe looked foolish and now I don’t care if I now look foolish or not (laughter)*. *And I think “I am who I am and if I make a mistake*, *so what*?*”* (P4G1)

#### Challenges of ageing

The negative aspects of ageing included participants’ existing medical conditions not allowing them to fully engage with their activity. Although age-related conditions, such as arthritis, made certain stages of creative activities problematic for our participants, they were able to find others who were keen to help, be it other class participants or tutors. This help was greatly appreciated and meant that the participants could carry on enjoying the activity instead of feeling excluded and dropping out due to the physical challenges. About half of the participants also reported problems with memory that were hindering their progress, especially in relation to language learning and following complicated and lengthy instructions.

*And clear to me that the other thing*, *which is memorising vocabulary*, *or memorising anything at this point is a different kind of game from how it used to be*. *I mean*, *it’s just much more difficult*. *And Gaelic maybe is not the place for me to start with trying to remember stuff (laughter)*. (P8G2)

Two participants also raised the practical issue of not being able to hear the tutor over other students, due to their own hearing problems but also owing to the physical layout of the learning space. School classrooms and libraries where many of the activities take place are not usually designed with older adults in mind, and this may hinder learning and result in a disappointing experience for those relying on hearing aids, for example.

### Differential effects of activities

Another theme consisted of the perceived impacts the specific activities had on participants’ health (including cognition) and wellbeing.

#### Benefits of activity

All participants mentioned something positive resulting from their participation in a new activity. Around half commented on the mental demands of their activities more generally, with a quarter of participants feeling the new activity made their “brains work hard”. Some noticed specific cognitive benefits saying learning a language made them “more alert” and “sharper”, with creative activities “challenging them on all fronts”, perhaps even more so than language classes. One participant especially appreciated making new friends and seeing their “diary filling up” and “breaking up the week”, which could sometimes be monotonous.

*I think that’s how I would feel*, *I think*, *I found the whole thing quite uplifting*. *Getting out and meeting people*. *And I enjoyed all that and I think it made me quite bouncy*, *sort of*, *a bit*, *you know*, *happy*, *different*? *So that’s what I think I felt with it all*. (P9G2)

Trying something new also resulted in more openness to doing more in the future. Some participants carried on with their new activity, others reported doing more of what they did before, and some were keen to try more new activities. About a third mentioned a new activity’s ability to expand their horizons. This was an interesting realisation for them, that even at an older age they can still find something novel and surprising. Instead of threatening their self-identity, this provided a sense of continuity (still knowing and doing what they like) but also an opportunity to grow in ways they did not expect.

*I think for me it was that saying “well*, *you know*, *the horizon’s bigger than just what you know and what you like*, *you could still do those things but there’s lots of other things out there*, *you know*.*”* (P8G1)

About two-thirds of participants appreciated the study giving them a new lease of life and making them realise that it is possible to “keep learning despite your age”. This could feel very empowering, knowing that their later life was an opportunity, and that they still have the right to do new things and experience growth.

*Most of us*, *and I’m probably one of the oldest*, *at our time of life an awful lot of your thoughts are about the past*. *But this taking part in this study we’re actually into doing something for the future*. *[…] So*, *you’re looking to the future*, *which is nice*, *quite unusual*, *novel*. *And it’s good*. *Because it means your life’s not done*, *you can*, *you’re still working at it and you’re still learning*, *so*, *that’s something I think is good*. (P5G1)

#### No change in abilities

When asked about any changes to their general wellbeing or mental abilities most participants did not say anything, with about a third specifically mentioning not noticing any changes.

Four participants even voiced their doubts about the activities having what it takes to benefit cognition. For example, they felt the courses were not long enough to produce any real changes or said they did not believe that creative activities offered anything to benefit cognitive abilities. Instead, they thought that perhaps language or computer classes would be better to “stimulate the brain more”. Those latter activities were seen as a more direct way to counteract cognitive decline, with participants perhaps considering crafts as leisurely play rather than the hard work required to learn a language, for example.

### General project feedback and considerations

This final theme explores participants’ opinions of the study and anything else they felt would be worth considering.

#### Opinions on activities and tutors

Opinions about the activities participants took up, the tutors, and the adult education classes overall were mostly positive, although not without criticism. While most participants had positive experiences with their classes and tutors and were keen to go back themselves or to recommend the class to friends, some were disappointed with their tutors, the structure of the courses, the difficult timing, or lack of provision.

*The teacher wasn’t that good*. *And that I think really was the problem because there was too much*, *it wasn’t enough structured learning for me*, *I found it quite boring*, *and of course I didn’t do enough homework*, *so that (laughter) because then it compounded*. (P2G3)

#### Challenges of adult education classes

Challenges of community-based classes recognised by most of our participants included the mixed ability nature of classes that were specifically described as for beginners or all levels, making it difficult to learn and get tutor’s attention and time. Participants also commented on the timing of some classes, which either took place on days when they were on “granny duty” or in the evening. Timing was especially important during the winter; activities finishing late in the evening brought up safety concerns, specifically for women. Those activities were often timed to accommodate younger people with work commitments, who cannot readily take part during the day, but this may inadvertently exclude older people from participating.

*The downside was it was winter*, *there were dark nights and I was* … *Find it difficult driving at night now and so I had to use public transport and it was 11 o’clock before I got home*. *And I just found that sort of took the edge off enjoying the class because I did enjoy the class but then having to*, *you know*, *walk in the dark*, *wait for the bus*, *get back*, *11 o’clock “oh time for bed”*. *That was the downside…* (P4G1)

Participants also talked about the practical challenges of the online booking system that opened from midnight and unless a potential student signed up as soon as the system allowed, they were unlikely to get a place on the more popular courses. Lastly, those who lived outside of Edinburgh faced the challenge of more limited provision or longer travel. For example, a participant who lives roughly 15 miles from Edinburgh city centre made that point when asked about activities available in their local area:

*Well*, *languages*, *probably*, *I refused to do that*, *computers… I refused but not so much the crafts*. *There’ll be crafts I’ve done*. *And I’ve done pottery*, *so it had to be a new thing*, *so*, *yeah but I should have travelled…* (P4G3)

#### Benefits of adult education classes

About a third of our participants recognised the positive aspects of local provision, including the existence of activities “for the less able”, awareness of locations and services they may need to use in the future, and the fact that for those who live in Edinburgh getting to their activity was relatively easy due to proximity to the nearest venue.

*I did silver jewellery making and I enjoyed it*, *it was great*, *the teacher was really good*, *very kind*. *There was one*, *it was a night class and I never take night classes but it was across the street from my flat*, *so I couldn’t really say that it was difficult to get there*. (P8G2)

#### Project feedback

To conclude the sessions, participants discussed the overall study structure including the activity allocation based on their previous exposure and location. Some participants were allocated to an activity they would never have considered, although in some cases they did come to enjoy it. This was a nice surprise and perhaps made them more open to trying something out of their comfort zone in the future. Although some participants wanted the encouragement to do something different, others said they would have rather avoided activities they had no interest in. This was especially true of bingo and social groups, perhaps because they are seen as stereotypical activities for those who are older and unable to participate in other activities and our participants did not see themselves this way.

The discussions also considered the type of person who normally volunteers for studies. About a third of our participants wondered whether those who do not generally want to “better themselves” would benefit more from taking part in the study because of their general isolation or unwillingness to try something new.

*I think we kind of knew in a way that this course was meant to stimulate you and […] you’re feeling you’re getting older (laughter) and you want to*, *sort of*, *break out of that*, *you know*, *“oh this might help to*, *sort of*, *rejuvenate you”*. *So*, *the idea that this was going to help in some way or other to rejuvenate*, *you know*, *challenge you*. *So*, *you know*, *I kind of approved of this whole exercise looking into what stops people from being older than they need to be*. (P3G1)

## Discussion and conclusions

Within a study examining community-based leisure activities as potential cognitive interventions for older adults, participants described their experiences of engagement positively. Many of the positives centred around enjoyment, meeting new people, and new learning boosting participants’ confidence and making them feel like there was still something they could find new and invigorating regardless of their age. The negative aspects generally concerned challenges specific to activities (e.g., handling specialist equipment or physical demands) and the fear and anxiety produced by doing something outside of one’s comfort zone. Participants also identified ageing as both a potential facilitator and a barrier to participation, with facilitators including greater time available after retirement, and barriers including age-related limitations (e.g., physical challenges). Although most participants did not notice any changes, a minority reported perceived gains in their general wellbeing and feeling “sharper”. Participants’ general project feedback included the positive and negative aspects of adult education classes, and opinions on study design. Participants also raised the issue that volunteers may differ from those who don’t seek out opportunities to improve.

Our findings are broadly in line with previous research examining facilitators and barriers to social participation and engagement in learning opportunities, including internal (e.g., enjoyment, lack of knowledge and confidence), external (e.g., costs, other commitments, provision) and social factors (e.g., socialisation, family/friend support) [16–20,30]. We note that our participants did engage in a new activity; much less is known about those who do not readily participate. The barriers and facilitators to engagement in leisure activities may be different for them compared to the more active individuals and should be explored along with potential greater health and wellbeing benefits in this group [12]. This understanding could then be used to encourage engagement of more sedentary individuals by targeting those specific factors.

Our participants spoke of their new experiences invigorating them and giving them something to look forward to, which for some was rare given a focus on looking back. They also highlighted the importance of enjoyment and focusing on what they wanted to do in their later years, consistent with SST [14]. Some of those engaging in activities initially not of interest came to enjoy them. However, others did not, resulting in them feeling they wasted their valued time. This may suggest that factors, such as the participants’ motivation, preconceptions about the activity [24] and their personality, such as openness to experience [7] may play an important role, and this should be explored further. This knowledge could then help better frame messages encouraging older adults to engage in specific activities.

The discussions also touched on negative stereotypes of ageing [21–23], including older people feeling inferior to their younger counterparts, and less eligible to try new things [24]. However, the very experience of trying something new, going outside of their comfort zone or conquering something that felt terrifying, such as specialist equipment [9], made some participants more confident and empowered. Some of our participants were keen to try something they previously thought was unattainable, such as art classes for those who were told they couldn’t draw at school. Others had been so affected by negative school experiences that the thought of doing this again was overwhelming [18]. Regardless, this study was a stepping-stone for many of our participants to challenge themselves in the future. Older adults should be encouraged to live a more engaged lifestyle, which could include trying new things, to benefit their general wellbeing [10,11].

Our results also provide some support for studies exploring the benefits of an engaged lifestyle and similar cognitive interventions designed in-house [3,8,9]. Some of our participants reported cognitive and health and wellbeing benefits, although others did not perceive any changes. This variation is likely due to the specific structure of learning and the directly comparable experience of all participants in those studies. Our participants often felt the community learning was not well-structured with no set curriculum, and consequently, their experiences differed depending on when and where they attended their classes. The aims of community-based classes are also different to those of interventions; emphasis is not on the progression of learning per se but enjoyment. To bridge this gap, provision of well-structured community-based activities for those specifically seeking improvement should be considered.

Our study was carried out in Edinburgh, Scotland, and it is likely that provision of activities may differ compared with other locations and cultures. From our own experience, provision became more limited outside of the city. Future studies should focus on other cities, towns and villages, as well as other countries, to allow comparisons with different localities and cultures. We chose to use activities already running in the community to break down the translational barrier often seen in interventions designed specifically for research. This was both an advantage and a hinderance. The activities used in this study are what people would normally experience when signing up to participate in a community-based activity, improving the study’s ecological validity. However, the unstandardised nature of those activities meant that participants’ experiences differed based on location and time, even for the same type of activity. Additionally, the provision of activities varied by location, which can hinder engagement [17,18]. Although some of our participants felt that they should have travelled to ensure a better pool of available activities, the focus should be on providing good quality activities close to older people, not making them travel further to participate.

In conclusion, our study considering the potential benefits of community-based leisure activities for older adults’ health and wellbeing was generally well received. Most focus group participants reported enjoying their new activity and being positively challenged, with some even perceiving specific benefits to their general wellbeing and cognition. Our participants also described the challenges of their specific activities as well as general barriers to taking up something new. Future studies should focus on other locations (in the UK and in other countries) to allow for comparison due to differences in provision and expectations of engagement in activities in later life. Barriers to engagement in leisure activities and the effects of an engaged lifestyle in less active individuals should be considered. It is important that older adults are able and encouraged to lead an engaged lifestyle, including participation in new and potentially beneficial activities. Local authorities, though challenged with resource allocation, should be supported to formalise the structure and provision of such activities to support older adults’ health and wellbeing.

## Supporting information

S1 Appendix Post-intervention focus group agenda. (DOCX)
