# Supplementary material for: Older adults’ experiences of taking up a new community-based leisure activity to promote brain health: A focus group study
Source: PLoS One. 2023 Sep 11;18(9):e0290623. doi: 10.1371/journal.pone.0290623 (PMC10495001; doi:10.1371/journal.pone.0290623)
Supplement: S1 Appendix — (DOCX) [file pone.0290623.s001.docx]

**S1 Appendix**

**Post-intervention Focus Group Agenda**

**Post-intervention Focus Group Agenda**

We are glad to have you here today. We would like to thank you for your time in our project looking at whether taking up a new activity is beneficial to thinking skills. Your participation has been very helpful to us and has given us information about how participation in a range of new activities may improve cognitive function and wellbeing in older adults.

There is an Information Sheet in front of you for you to read and a consent form for you to sign. Please feel free to ask me any questions you may have before you sign anything. This session will be video and audio recorded. Those recordings will be used for transcribing only; they will not be available to anyone else and will not be published. You do not need to say your name before you speak but if you do, the transcript will not contain that information. You will be allocated a number, such as Participant 1, Group 1 and your responses will be transcribed in this fashion throughout.

Today, we want to hear your thoughts about the experience of taking up a new activity. We will talk about things like: what you liked and what you did not like about your new activity; what you thought was particularly useful, and what you thought did not really help you. Let’s get started.

1. First, let’s talk about the activities overall. Some of you have taken up two new activities as part of this project but for now let’s focus on your first activity. Tell me about the things you learned from your initial new activity. [take activities in turn: **1. Physical activity; 2. Social group; 3. Computer; 4. Language; 5. Creative (for example woodwork, pottery, stained glass, printmaking)**].

2. What did you like most about your initial allocated activity?

3. What did you find challenging about that activity?

a. How were you able to overcome those challenges?

4. Was there anything you did not like about your first activity?

a. What made you dislike those things?

5. How did you find keeping up your attendance?

*Probes*

- *What was your motivation to keep attending the classes?*

6. For those of you who were allocated to a second activity, what did you learn by taking part in your second activity?

7. What did you like most about your second activity?

8. What did you find challenging about the second allocated activity?

a. How were you able to overcome those challenges?

9. Was there anything you did not like about your second activity?

a. What made you dislike those things?

10. Did your second activity change your motivation and experience of keeping up your attendance?

11. I now want to hear from all of you. What do you think are the benefits of taking up a new activity?

12. What do you think are the challenges of taking up a new activity?

13. Do you think that taking up a new activity improved your mental abilities?

a. If yes, could you give examples to illustrate your point?

b. For those of you who took up two activities, did you observe any differences in your mental abilities between taking part in the two activities?

14. Do you think that taking up a new activity was helpful in improving your general health and wellbeing?

*Probes*

- *Do you think that aspects of your general health and wellbeing (such as sleep, fitness, diet, social networking) improved by taking up your first new activity?*

a. For those of you who did a second activity, did you observe any differences in your general health and wellbeing between taking part in the two activities?

15. Was your allocated activity (first or second) something you would have chosen for yourself or was it something you never thought of taking up?

16. Since completing your participation in the project, have you carried on with your allocated activity/activities?

a. Have any of you taken up something different?

17. Are there any other activities you would like to try?

18. Finally, is there anything else about your experience with taking up a new activity that you would like to share?
